# Structural properties of the reconciliation space and their applications in enumerating nearly-optimal reconciliations between a gene tree and a species tree

**DOI:** 10.1186/1471-2105-12-S9-S7

**Published:** 2011-10-05

**Authors:** Taoyang Wu, Louxin Zhang

**Affiliations:** 1 Department of Mathematics, National University of Singapore, Singapore 119076

## Abstract

**Introduction:**

A gene tree for a gene family is often discordant with the containing species tree because of its complex evolutionary course during which gene duplication, gene loss and incomplete lineage sorting events might occur. Hence, it is of great challenge to infer the containing species tree from a set of gene trees. One common approach to this inference problem is through gene tree and species tree reconciliation.

**Results:**

In this paper, we generalize the traditional least common ancestor (LCA) reconciliation to define a reconciliation between a gene tree and species tree under the tree homomorphism framework. We then study the structural properties of the space of all reconciliations between a gene tree and a species tree in terms of the gene duplication, gene loss or deep coalescence costs. As application, we show that the LCA reconciliation is the unique one that has the minimum deep coalescence cost, provide a novel characterization of the reconciliations with the optimal duplication cost, and present efficient algorithms for enumerating (nearly-)optimal reconciliations with respect to each cost.

**Conclusions:**

This work provides a new graph-theoretic framework for studying gene tree and species tree reconciliations.

## Background

With much higher speed than the traditional Sanger sequencing technology, the ultra-deep sequencing technology has made huge amounts of molecular data available for genomics study [1]. It provides an unprecedented opportunity to infer phylogenetic trees from multilocus and genomics data. One approach to inferring phylogeny from multilocus data is to reconstruct a gene tree from each locus and then to combine the resulting trees into a phylogeny, called the containing species tree. Gene trees are often different since each gene family might undergo different mutational events such as gene duplication and loss, horizontal gene transfer, and incomplete lineage sorting [2,3]. Therefore, the containing species tree is inferred from gene trees by reconciling it with each gene tree to minimize the total number of hypothetical evolutionary events that are responsible for the discordance between the trees.

The gene tree and species tree reconciliation was first introduced by Goodman et al. [4] and formally defined by Page [5]. Given a gene tree for a gene family and a containing species tree, a reconciliation between them represents an evolutionary scenario of the gene family within the evolutionary history represented by the species tree [4]. To study gene duplication history, gene tree and species tree are reconciled to minimize the number of gene duplications and/or losses. The mathematical and algorithmic issues of gene tree and species tree reconciliations have been intensively studied in the past decade [6-14]. For example, it has been shown that the so-called least common ancestor (LCA) reconciliation has the minimum duplication and loss cost [9,15].

Although the LCA reconciliation is optimal in terms of the duplication cost, it may not represent the true evolution of the gene family being considered. Indeed, recent studies suggest that more than one reconciliations may occur with the highest probability [16,17]. Such studies [3,14,17,18] in the stochastic framework assume that the discordance between a gene tree and a species tree is caused by incomplete lineage sorting and adopt Kingman’s coalescent theory from population genetics [19].

The fact that the LCA reconciliation may not be the unique optimal with respect to the duplication cost motivates researchers to study the space of all the reconciliations and develop algorithms to enumerate nearly-optimal reconciliations for a species tree and a gene tree [20,21]. In this paper, we take a different approach to these two issues. We generalize the LCA reconciliation to define an arbitrary reconciliation as a vertex-mapping from a gene tree to a species tree that preserves the hierarchical structure of the gene tree. Our approach is essentially different from the existing ones [20,22], where the specific mutation events are used and a gene tree vertex is mapped to a species tree branch to specify a duplication event. One advantage of our approach over the others is that we separate reconciliation concept from the cost models that are used to measure the tree discordance. Because of this, we are able to study the structural properties of the space of all reconciliations between a gene tree and a species tree in the same manner for each of the three cost models. We show that the LCA reconciliation has not only the minimum duplication and loss cost [9,15], but also the minimum deep coalescence cost. We also present a novel characterization of the reconciliations with the optimal duplication cost, and develop efficient algorithms for enumerating (nearly-)optimal reconciliations with respect to each cost model.

## Methods

### Basic notations

Species evolve from their common ancestor through a series of speciation events. A species tree represents the evolutionary history of a set of species. A gene family might evolve from its common ancestral gene through gene duplication and loss events. Here we will assume that no lateral gene transfer has occurred.

Both gene and species trees are rooted trees with labeled leaves. In a species tree, a leaf *x* represents a species, the label of *x*. Hence, the species tree is uniquely leaf-labeled. In a gene tree, a leaf *y* represents a gene found in a species. To infer the duplication history of a gene family, its gene tree and the containing species tree is reconciled [4]. For this purpose, a leaf of a gene tree is labeled with the containing species. Since a species may contain duplicate genes, two leaves in a gene tree can have the same label.

Let *T* be a species or gene tree; its vertex set and edge set are denoted by *V*(*T*) and *E*(*T*), respectively. Given two vertices *u* and *v* in *T*, there exists a unique path *P*(*u*, *v*) from *u* to *v*. The number of edges in *P*(*u*, *v*), denoted by *d*(*u*, *v*), is called the *distance* between *u* and *v*. Note that *d*(*u*, *v*) = 0 if and only if *u* = *v*. The node *v* is a *descendant* of *u* or *u* is an *ancestor* of *v*, denoted by *v* ≤ *u*, if *u* is on the unique path from *r*(*T*), the root of *T*, to v. For simplicity, we also write *v* <*u* if *v* ≤ *u* and *v* ≠ *u*. Given a set *A* of vertices in *T*, *u* is a *common ancestor* of *A* if and only if *v* ≤ *u* for every *v* ∈ *A*. In addition, if *u* ≤ *u′* for any other common ancestor *u′* of *A*, then we say *u* is the *least common ancestor* of *A*, written as lca(*A*), or lca(*u*_1_, ⋯, *u_k_*) if *A* = {*u*_1_, ⋯, *u_k_*}.

For each vertex *u* in *T* with *u* ≠ *r*(*T*), the *parent* of *u*, denoted by *p*(*u*), is the unique vertex in *T* that is adjacent to *u* and contained on the path from *r*(*T*) to *u*. In this case, *u* is also called a *child* of *p*(*u*). The out-degree of *u*, denoted by *d*(*u*), is defined as the number of the children of *u*. Obviously, a node is a leaf if and only if its out-degree is 0. Non-leaf nodes are internal nodes; they form a subset *V°*(*T*) of *V*(*T*). If every internal vertex has out-degree two, then *T* is *binary*. For an internal vertex *u* in a binary tree, its two children are denoted by *u*_1_ and *u*_2_, unless stated otherwise. In this study, we will focus on the case that gene trees and species trees are binary. For a vertex *u*, we use *L*(*u*) to denote the set of the labels of its leaf descendants and call it the *cluster* induced by *u*. Finally, we use *L*(*T*) to denote the set of leaf labels, i.e., the cluster induced by the root of *T*.

### Reconciliation between gene tree and species tree

Let *S* be a species tree over a set of species and *G* a gene tree such that *L*(*G*) ⊆ *L*(*S*), i.e., *G* is over all the homologous genes of a gene family found in some species. A map *f* from *V*(*G*) to *V*(*S*) is order-preserving if for each pair of vertices *u*, *v* in *G*, *u* ≤ *v* implies *f*(*u*) ≤ *f*(*v*)*;* it is leaf-preserving if, for each leaf *x* in *G*, *f*(*x*) is the unique leaf in *S* that has the same label.

A *reconciliation* between a gene tree *G* and a species tree *S* is a leaf-preserving and order-preserving map from *V*(*G*) to *V*(*S*). Clearly, a reconciliation *f* between *G* and *S* is necessarily an inclusion-preserving mapping (see [8]), that is, for each pair of vertices *u*, *v* in *G*, *u* ≤ *v* implies *L*(*f*(*u*)) ⊆ *L*(*f*(*v*)). However, the reverse statement is not true. For instance, the mapping that maps each vertex of *G* to the root of *S* is an inclusion-preserving mapping, but according to our definition, it is not leaf-preserving, and hence not a reconciliation.

Note that our definition is consistent with the one used in [20], where a reconciliation is defined as a mapping from *V*(*G*) to *V*(*S*) ∪ *E*(*S*) that satisfies three constraints: base constraint, tree mapping constraint and ancestor consistency constraint. Roughly speaking, our order-preserving condition corresponds to the ancestor consistency constraint, and the leaf-preserving condition is related to the base constraint, while the tree mapaaping constraint is not needed in our setting. The main difference between these two frameworks is the model used to interpret mappings. For example, in [20], a duplication event is associated to a vertex *v* in *G* if and only if *v* is mapped to an edge, while in our model, whether *v* is associated with a duplication event is not solely determined by the image of *v*.

A reconciliation represents a hypothetical evolutionary history of the gene family. In a gene tree, an internal vertex *u* represents the common ancestor of the genes represented by the leaves below it. The property just reflects the intuitive fact that *u* is an ancient gene appearing in some common ancestor of the species from which the genes are taken. Recall that in species tree each branch represents an ancestral species. Under the reconciliation *f*, we considered *u* as the gene ancestor found in the species represented by the branch entering *f*(*u*).

There is a canonical partial order ≼ on the set of reconciliations between *G* and *S*: for any *f′* and *f*, *f*′ ≼ *f* if and only if *f′*(*v*) ≤ *f*(*v*) holds for every vertex *v* in *G*. Define a mapping *M* from *G* to *S* recursively as:

*M* is called the *least common ancestor* (LCA) reconciliation between *G* and *S*. Note that we have *M* ≼ *f* for every reconciliation *f* between *G* and *S*, because it is easy to see that *M*(*u*) ≤ *f*(*u*) holds for all *u* ∈ *V*(*G*), by a bottom-up traversal.

### Inference of gene duplications

**Figure 1:**
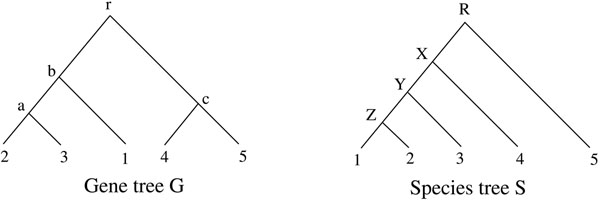


**Figure 2:**
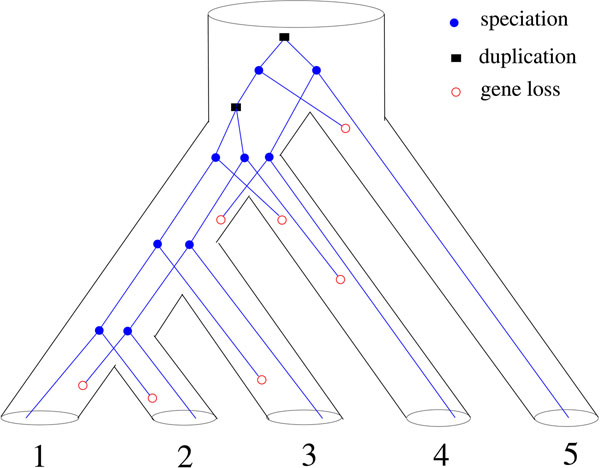


If the discord of a gene tree *G* and its containing species tree *S* is due to gene duplication, a reconciliation *f* between them represents a plausible duplication history of genes. For an internal vertex *u*, a *duplication event* is associated with *u* if and only if one of the following two conditions holds: (D-i) *f*(*u*) = *f*(*u*_1_), *f*(*u*) = *f*(*u*_2_) or both hold; (D-ii) *P*(*f*(*u*), *f*(*u*_1_)) and *P*(*f*(*u*), *f*(*u*_2_)) contain a common edge. In the literature (see [5]), when the LCA reconciliation *M* is used for inferring gene duplications, the duplication condition used is (D-i). This is correct for the LCA reconciliation between a gene tree and a species tree. However, this stringent condition is no longer appropriate as the definition of duplication events for arbitrary reconciliations. For example, consider the reconciliation *f* between the gene tree *G* and the species tree *S* as in Figure 1. If the original definition is used, as proposed in [8], only one duplication is inferred, which is associated with *r*. However, one duplication cannot produce such a gene family having the gene tree *G*. On the other hand, if our proposed definition is used, two duplications are inferred, one associated with *r* and the other with *b*; the implied duplication scenario is given in Figure 2.

Now, for an internal node *u*, we let *δ_f_*(*u*) = 1 if there is a duplication event associated with it, and *δ_f_*(*u*) = 0 otherwise. Then the *gene duplication* cost gd (*f*) of *f* is defined as:(1)

### Gene loss cost

Let *G* be a gene tree, *S* a species tree and *f* a reconciliation between *G* and *S*. Then the *number of losses l_f_*(*u*) associated to an internal vertex *u* is defined as:

Note that our definition of *l_f_*(*u*) is a generalization of the one introduced by Ma et al. in [6], and is consistent with the one in [20]. When *f* is the LCA reconciliation, our definition agrees with the traditional one [5,6]. For later use, it is often convenient to combine the two formulae in the above definition, i.e., we have:(2)

For simplicity, we also set *l_f_*(*x*) = 0 for any leaf *x* of *G*. The *gene loss* cost gl(*f*) of *f* is defined as:(3)

For example, for the reconciliation *f* in Figure 1, we have gl(*f*) = 7 by noting that:

which can also been observed from Figure 2.

### Deep coalescence cost

If the discord of a gene tree *G* and a species tree *S* is due to incomplete lineage sorting, a reconciliation *f* between them is measured by the deep coalescence cost [3]. Given a branch *e* in *S*, we say that there are *k* (*k* > 0) *extra lineages* (with respect to *f*) failing to coalesce on *e*, denoted by τ*_f_*(*e*) = *k*, if there exist *k* + 1 distinct edges (*u_i_*, *v_i_*) (1 ≤ *i* ≤ *k* + 1) in *G* such that *e* is on the path *P*(*f*(*u_i_*), *f*(*v_i_*)) for each *i;* otherwise, we let *τ_f_*(*e*) = 0. The *deep coalescence* cost dc(*f*) of *f* is then defined as:

i.e., the total number of the extra lineages with respect to *f* on all branches of *S*. For example, for the reconciliation *f* in Figure 1, we have dc(*f*) = 3 by noting that:

## Results

### The monotonicity of the reconciliation costs

We first have the following useful observations on the gene duplication cost.

**Lemma 1*** Let f be a reconciliation between a gene tree G and a species tree S. If u is an internal vertex in G with children u*_1_* and u*_2_, *then the following observations hold*.

*(i): δ_f_*(*u*) = 1 *if and only if f*(*u*) ∈ {*f*(*u*_1_), *f*(*u*_2_)} *or lca*(*f*(*u*_1_), *f*(*u*_2_)) <*f*(*u*).

*(ii): δ_f_*(*u*) = 0 *if and only if f*(*u*_1_) ≠ *f*(*u*) ≠ *f*(*u*_2_) *and lca*(*f*(*u*_1_),*f*(*u*_2_)) = *f*(*u*).

*(iii): If L*(*f*(*u*_1_)) ∩ *L*(*f*(*u*_2_)) ≠ ∅, *then δ_f_*(*u*) = 1.

*(iv): If f*(*u*) >*M*(*u*), *then δ_f_*(*u*) = 1.

*(v): If δ_f_*(*u*) = 0, *then f*(*u*) = *M*(*u*) *and L*(*f*(*u*_1_)) ∩ *L*(*f*(*u*_2_)) = ∅.

*Proof:* Since *δ_f_*(*u*) is either 0 or 1, (ii) clearly follows from (i), and (v) follows from (iii) and (iv).

To establish (i), it suffices to show that lca(*f*(*u*_1_), *f*(*u*_2_)) <*f*(*u*) if and only if *P*(*f*(*u*), *f*(*u*_1_)) and *P*(*f*(*u*),*f*(*u*_2_)) share a common edge. Indeed, if we have lca(*f*(*u*_1_),*f*(*u*_2_)) <*f*(*u*), then *P*(*f*(*u*), *f*(*u*_1_)) and *P*(*f*(*u*), *f*(*u*_2_)) share the edge that is incident to *lc*a(*f*(*u*_1_), *f*(*u*_2_)) and its parent. On the other hand, if *P*(*f*(*u*), *f*(*u*_1_)) and *P*(*f*(*u*), *f*(*u*_2_)) share a common edge (*s*, *s′*) with *s′* <*s*, then *s′* is a common ancestor of *f*(*u*_1_) and *f*(*u*_2_) such that *s′* <*s* ≤ *f*(*u*). Therefore we have *L*(*f*(*u*_1_), *f*(*u*_2_)) ≤ *s′* <*f*(*u*), as required.

Now we proceed to prove (iii). If *L*(*f*(*u*_1_)) ∩ *L*(*f*(*u*_2_)) ≠ ∅, then we have either *f*(*u*_1_) ≤ *f*(*u*_2_) or *f*(*u*_2_) ≤ *f*(*u*_1_). By symmetry, we may assume *f*(*u*_1_) ≤ *f*(*u*_2_), and hence lca(*f*(*u*_1_), *f*(*u*_2_)) = *f*(*u*_2_) holds. Now there are two cases to be considered, i.e., *f*(u_2_) = *f*(*u*) and *f*(u_2_) <*f*(*u*). By (i), we can conclude *δ_f_*(*u*) = 1 in both of them.

It remains to show (iv). Note first that we can assume lc*a*(*f*(*u*_1_), *f*(*u*_2_)) >*M*(*u*), because otherwise we have lc*a*( *f*(*u*_1_), *f*(*u*_2_)) = *M*(*u*) <*f*(*u*), and hence *δ_f_*(*u*) = 1 by (i). It follows that *f*(*u_i_*) >*M*(*u*) for some *i* = 1, 2. Therefore, by switching *u*_1_ and *u*_2_ if necessary, we can further assume *f*(*u*_1_) >*M*(*u*). Now we need to consider two cases: *f*(*u*_2_) >*M*(*u*) and *f*(*u*_2_) ≤ *M*(*u*). If *f*(*u*_2_) >*M*(*u*), then *f*(*u*_1_) and *f*(*u*_2_) are both contained in the path *P*(*f*(*u*), *M*(*u*)), and thus *L*(*f*(*u*_1_)) ∩ *L*(*f*(*u*_2_)) ≠ ∅ holds. On the other hand, *f*(*u*_2_) ≤ *M*(*u*) implies *f*(*u*_2_) ≤ *f*(*u*_1_), and hence also *L*(*f*(*u*_1_)) ∩ *L*(*f*(*u*_2_)) ≠ ∅. Since in both cases we have *L*(*f*(*u*_1_)) ∩ *L*(*f*(*u*_2_)) = ∅, by (iii) we obtain *δ_f_*(*u*) ≠ 1, as required. Q.E.D

Note that (i) in the above lemma provides an additional characterization of gene duplication events. This characterization is easier for calculation while the original definition is more natural, from an evolutionary point of view. By (v) in the above lemma, if a speciation event happens at *u*, i.e., *δ_f_*(*u*) = 0, then we have *f*(*u*) = *M*(*u*). This agrees with the definition of reconciliation in [20]. Now we have the following main result.

**Theorem 2*** Let f and f′ be two distinct reconciliations between a gene tree G and a species tree S with f′* ≼ *f; then we have:*(4)

*In addition*, *δ_f′_*(*u*) ≤ *δ_f_*(*u*) *for each u* ∈ *V*(*G*), *where the equality holds for each u* ∈ *V*(*G*) *if and only if* gd(*f′*) = gd(*f*).

*Proof:* Let *D*(*f′*, *f*) be the number of vertices *v* in *V*(*G*) with *f*(*v*) ≠ *f′*(*v*)*;* then the following observation plays an important role in our proof of the theorem.

**Lemma 3** Let f and f′ be the two reconciliations as given in the theorem. Then there exists a reconciliation f* between G and S that satisfies the following three conditions:

*Proof:* To establish the above lemma, we select a minimal element *v*_min_ (with respect to the partial order ≤ on *V*(*G*)) in the set {*u* ∈ *V*(*G*) : *f*(*u*) ≠ *f′*(*u*)}, which is necessarily non-empty by the assumption *f* ≠ *f′*. In other words, *f*(*v*) = *f′*(*v*) holds for any *v* such that *v* <*v*_min_. Now consider the map f* defined as:

Then f* is a reconciliation between *G* and *S*. To see this, note first that *f* and *f′* are reconciliations, and hence they are leaf-preserving. Therefore we know f* is also leaf-preserving. Let *u* and *v* be a pair of vertices in *G* with *u* ≤ *v*. If *u*, *v* ∈ *V*(*G*) *–* {*v*_min_}, then f*(*u*) ≤ f*(*v*) because *f* is order-preserving. On the other hand, if *u* = *v*_min_ and *v* ≠ *u*, then we also have:

where we use the fact that *f′* is order-preserving and *f′* ≼ *f* in the first and second inequality, respectively.

Finally, suppose *v* = *v*_min_ and *u* ≠ *v*. By the way that *v*_min_ is chosen, we have *f′*(*u*) = *f*(*u*), and hence also:

This shows f* is order-preserving, and hence f* is indeed a reconciliation between *G* and *S*.

It remains to show that f* satisfies the three conditions required in the claim. Since *f′* ≼ *f*, from the construction of f* we have *f′* ≼ f* ≼ *f*. Noting that *v*_min_ is the only vertex in *V*(*G*) that is mapped to different images by *f* and f*, we have *D*(f*, *f*) = 1. Finally, for any *v* in *G*, *f′*(*v*) ≠ f*(*v*) if and only if *v* ≠ *v*_min_ and *f′*(*v*) ≠ *f*(*v*). In other words, we have *D*(*f′*, f*) = *D*(*f′*, *f*) – 1, which completes the proof of Lemma 3. Q.E.D.

Now it suffices to prove the theorem for the special case *D*(*f*, *f′*) = 1. Indeed, if *D*(*f*, *f′*) = *m* > 1, then by Lemma 3, there exist *m* +1 reconciliations *f*_1_ := *f′*, *f*_2_, *⋯*, *f_m_*_+1_ := *f* so that *f_i_* ≼ *f_i_*_+1_ and *D*(*f_i_*, *f_i_*_+1_) = 1 for 1 ≤ *i* ≤ *m*. Applying the theorem (in the special case mentioned above) for each pair of reconciliations *fi* and *f_i_*_+1_, we have gd(*f_i_*) ≤ gd(*f_i_*_+1_) for 1 ≤ *i* ≤ *m*, and hence gd(*f′*) = gd(*f*_1_) ≤ gd(*f_m_*_+1_) = gd(*f*). Similarly, we can show gl(*f′*) < gl(*f*), dc(*f′*) < dc(*f*), and *δ_f′_*(*u*) ≤ *δ_f_*(*u*) for each *u* ∈ *V*(*G*), among which the last one implies that gd(*f*) = gd(*f′*) if and only if *δ_f_*(*u*) = *δ_f′_*(*u*) for each *u* ∈ *V*(*G*).

Now let *v* be the unique vertex in *G* with *f*(*v*) ≠ *f′*(*v*). Clearly, *v* is an internal vertex. If *v* is not the root, let *v*_0_ := *p*(*v*) be its parent and *v*_3_ be its sibling, that is, the other child of *v*_0_. The remainder argument will be divided into three cases, according to the cost measure considered.

### Duplication cost case

Noting that *f*(*v_i_*) = *f′*(*v_i_*) ≤ *f′*(*v*) <*f*(*v*) for *i* = 1, 2, we have:

By (i) in Lemma 1, this shows *δ_f_*(*v*) = 1, and hence *δ_f_*(*v*) ≥ *δ_f′_*(*v*). If *v* is the root of *G*, then we have gd(*f*) – gd(*f′*) = *δ_f_*(*v*) *– δ_f′_*(*v*) ≥ 0, as required.

Now we assume *v* is not the root, and proceed to show *δ_f_*(*v*_0_) ≥ *δ_f′_*(*v*_0_). To begin with, we can assume *δ_f′ _*(*v*_0_) = 1, because otherwise the inequality trivially holds. In addition, we can further assume *f*(*v*) <*f*(*v*_0_) and *f*(*v*_3_) <*f*(*v*_0_), because otherwise we have *δ_f_*(*v*_0_) = 1, which also implies the inequality. It follows that we have:

By (i) in Lemma 1, this leads to lca(*f′*(*v*), *f′*(*v*_3_)) <*f′*(*v*_0_) = *f*(*v*_0_). Let *s* be the child of *f*(*v*_0_) so that lca(*f′*(*v*), *f′*(*v*_3_)) ≤ s. Since *f′*(*v*) ≤ *f*(*v*) <*f*(*v*_0_) and *f′*(*v*_3_) = *f*(*v*_3_), *s* is also a common ancestor of *f*(*v*) and *f* (*v*_3_). Therefore we have lca (*f′* (*v*), *f′* (*v*_3_)) ≤ s <*f*(*v*_0_). Using (i) in Lemma 1 again, we can conclude *δ_f_*(*v*_0_) = 1, as required.

Since *v* is the only vertex in *G* with *f*(*v*) ≠ *f′*(*v*), for each internal vertex *g* ∈ *V*(*G*) *–* {*v*, *v*_0_} and its two children *g*_1_ and *g*_2_, we have:

By definition, this implies  for all *g* ∈ *V*(*G*) *–* {*v*, *v*_0_}. Combining the above observations, we can conclude that *δ_f_*(*u*) ≥ *δ_f′_*(*u*) for each *u* ∈ *V*(*G*). This leads to gd(*f*) ≥ gd(*f′*), where the equality holds if and only if *δ_f_*(*u*) = *δ_f′_*(*u*) for each *u* ∈ *V*(*G*).

### Gene loss case

Since *f*(*v_i_*) ≤ *f′*(*v*) <*f*(*v*) holds for *i* = 1,2, we have:(5)

Together with the definition of *l_f_*, we obtain:

Since *δ_f_*(*v*) ≥ *δ_f′_*(*v*), following the proof of the duplication cost case, and *d*(*f*(*v*), *f′*(*v*)) > 0, we can conclude that *l_f_*(*v*) *– l_f′_*(*v*) ≥ 0. If *v* is the root of *G*, then this leads to gl(*f*) > gl(*f′*), as required.

Now we assume *v* is not the root of *G*. Then we have:

where we use the observation that *f′*(*v*) <*f*(*v*) ≤ *f*(*v*_0_) implies:

Combining these results, we have:

Since gd(*f*) ≥ gd(*f′*), following the proof of the duplication cost case, and *d*(*f*(*v*), *f′*(*v*)) > 0, we obtain gl(*f*) > gl(*f′*), which completes the proof of this case.

### Deep coalescence case

Let *E_f_*(*S*) be the set of edges *e* in *S* such that there exists an edge (*u*,*u′*) in *G* such that *e* is contained in the directed path from *f*(*u*) to *f*(*u′*). Now by counting extra lineages in terms of the edges contained in paths that have form *P*(*f*(*u*), *f*(*u′*)) for some edge (*u*, *u′*) in *G*, we have:(6)

Since *E_f_*(*S*) = *E_f′_*(*S*) and *f*(*u*) = *f′*(*u*) for *u* ≠ *v*, the above formula implies:

if *v* is not the root of *G*. Here in the second equality we use the observation that *f*(*v*) is on the directed path from *f*(*v*_0_) to *f′*(*v*), and for *i* = 1,2, *f′*(*v*) is on the directed path from *f*(*v*) to *f*(*v_i_*). If *v* is the root of *G*, then a similar argument leads to:

which completes the proof. Q.E.D.

Since the LCA reconciliation is the minimal element in the space of reconciliations, the above theorem leads directly to the following result.

**Corollary 4*** Among all reconciliations between a gene tree G and a species tree S*, *the LCA reconciliation has* (*a*) *the minimum gene duplication cost*[9], (*b*) *the unique one with the optimal gene loss cost*[15]*and the optimal deep coalescence cost*.

Note that there is a close relationship among the gene duplication, gene loss and deep coalescence costs [7]. From their relationship, one can easily obtained the fact that the LCA is the unique one with the optimal gene loss cost from that it is the unique one with the optimal deep coalescence, but the reverse is not clear.

### Gd-optimal reconciliations

By Corollary 4, the LCA reconciliation is the unique optimal reconciliation for the gene loss cost, as well as the deep coalescence cost. However, the LCA reconciliation may not be the unique optimal one for the gene duplication cost (see [15]). For example, for the reconciliation *f* in Figure 1 and the LCA reconciliation M between the gene tree and species tree in Figure 1, we have gd(*f*) = gd(*M*) = 2. Since the reconciliations with the minimum gene duplication cost, which we shall refer to as *gd-optimal reconciliations*, may not be unique, in this section we will present a characterization of them, using the theoretical results developed above.

By Theorem 2, a reconciliation *f* is gd-optimal if and only if *δ_f_*(*u*) = *δ_M_*(*u*) holds for each vertex *u* in *G*. Based on it, we will show that there exists a unique maximal gd-optimal reconciliation M* so that *f* is gd-optimal if and only if *f* ≼ M* holds. The reconciliation M* between a gene tree *G* and a species tree *S* can be constructed as follows. For all *u* ∈ *V*(*G*) with *δ_M_*(*u*) = 0, M* maps *u* to *M*(*u*), i.e., M*(*u*) = *M*(*u*). For those *u* ∈ *V*(*G*) with *δ_M_*(*u*) = 1, we shall define M*(*u*) recursively. If *u* = *r*(*G*), i.e., it is the root of *G*, then M*(*u*) is defined as *r*(*S*), the root of *S*. Otherwise, M*(*p*(*u*)) has been defined, and M*(*u*) is defined as:

If *u* is a vertex in *G* such that *u* ≠ *r*(*G*), then *δ_M_*(*p*(*u*)) = 0 implies *M*(*u*) <*M*(*p*(*u*)) ≤ M*(*p*(*u*)), hence the mapping M* is well defined. In addition, M* is also a reconciliation between *G* and *S*. To see this, note that if *u* is a leaf in *G*, then we have *δ_M_*(*u*) = 0, which implies M*(*u*) = *M*(*u*) and hence M* is leaf-preserving. On the other hand, by the construction of M*, it is order-preserving. For example, for the gene tree and species tree in Figure 1, the reconciliation M* is defined as:

In this example, it is not difficult to check that gd(*f*) = gd(M*) holds for all *f* ≼ M*, which also follows directly from the following general result.

**Theorem 5*** Given a gene tree G and a species tree S*, *a reconciliation f is gd-optimal if and only if M* ≼ *f* ≼ M* *holds. In particular*, M* is the unique maximal gd-optimal reconciliation between G and S.

*Proof:* We need only to show that gd(*f*) = gd(*M*) for a reconciliation *f* if and only if *M* ≼ *f* ≼ M* holds, because this implies M* is indeed the unique maximal gd-optimal reconciliation.

To show that gd(*M*) = gd(*f*) holds for every reconciliation *f* with *M* ≼ *f* ≼ M*, it suffices to prove gd(M*) = gd(*M*), because together with Theorem 2, this implies gd(*f*) = gd(*M*) = gd(M*). To this end, we need only to show *δ_M_*(*u*) = δ_M*_(*u*) for each internal vertex *u* in *G*. Now fix an internal vertex *u* in *G*. Since *M* ≼ M*, we have δ_M*_(*u*) ≥ *δ_M_*(*u*) by Theorem 2. If *δ_M_*(*u*) = 1, then we have δ_M*_(*u*) = 1 = *δ_M_*(*u*). Therefore it remains to consider the case *δ_M_*(*u*) = 0. By (ii) in Lemma 1, *δ_M_*(*u*) = 0 implies *M*(*u*_1_) ≠ *M*(*u*) ≠ *M*(*u*_2_). Together with the construction of M*, we have M*(*u*_1_) ≠ M*(*u*) ≠ M*(*u*_2_). Since *M*(*u_i_*) ≤ M*(*u_i_*) ≤ M*(*u*) for *i* = 1, 2, we have:

By the construction of *M*, we know lca(*M*(*u*_1_), *M*(*u*_2_)) = *M*(*u*), and hence:

By (ii) in Lemma 1, this shows δ_M__*_ (*u*) = 0, as required.

To establish the other direction, assume gd(*f*) = gd(*M*) for a reconciliation *f*, and we shall show *f* ≼ M*, i.e., *f*(*u*) ≤ M*(*u*) for each internal *u* in *V*(*G*). To this end, fix an internal vertex *u* in *G*, and denote its two children by *u*_1_ and *u*_2_. If *δ_f_*(*u*) = 0, then by (v) in Lemma 1 we have *f*(*u*) = *M*(*u*), and hence *f*(*u*) = M*(*u*). Therefore, it remains to prove *f*(*u*) ≤ M*(*u*) for *δ_f_*(*u*) = 1, which will be established by induction. The base case is *u* being the root of *G;* then M*(*u*) is the root of *S*, and *f*(*u*) ≤ M*(*u*) trivially holds. For the induction step, let *u*_0_ := *p*(*u*) be the parent of *u;* then the induction assumption is *f*(*u*_0_) ≤ M*(*u*_0_). Now if *δ_M_*(*u*_0_) = 1, then by the definition of M* we have:

Otherwise, we have *δ_M_*(*u*_0_) = 0. Together with *M* ≼ *f*, *M* ≼ M* and gd(*M*) = gd(*f*) = gd(*M*), this leads to *δ_f_*(*u*_0_) = δ_M*_(*u*_0_) = 0 by Theorem 2. In view of (v) in Lemma 1, we obtain *M*(*u*_0_) = *f*(*u*_0_) = M*(*u*_0_). Since *δ_f_*(*u*_0_) = 0, (ii) in Lemma 1 implies *f*(*u*) <*f*(*u*_0_), and hence also:

By definition, M*(*u*) is the largest vertex in the set {*s* : *M*(*u*) ≤ *s* <M*(*u*_0_)}. Since M*(*u*_0_) = *M*(*u*_0_), we can conclude *f*(*u*) ≤ M*(*u*), which completes the proof. Q.E.D.

### Enumerate nearly-optimal reconciliations

Recall that there are other reconciliations having the minimum duplication cost than the LCA reconciliation. Moreover, in a biological study, a nearly-optimal reconciliation could be the correct solution to its problem. Therefore, it is of interest to study the following problem [20]: Given a positive number *ε*, compute the set of *nearly-optimal* reconciliations that have the duplication cost less than or equal to gd(*M*) + *ε*, where gd(*M*) is the minimum duplication cost a reconciliation between the gene tree and the species tree can have. Such a subset of the nearly-optimal reconciliations is denoted by Γ*_ε_*(*G*, *S*, gd), which is also a subset of Γ(*G*, *S*), the set of all reconciliations between *G* and *S*.

In this section we will present an algorithm for enumerating Γ*_ε_*(*G*, *S*, gd). To this end, we need to introduce some additional definitions. Following [20], for a vertex *u* ∈ *V*(*G*), let id(*u*) be the number of vertices that precede *u* according to the prefix traversal of *G*, where the left child *u*_1_ of a vertex *u* ∈ *V°*(*G*) is visited before the right child *u*_2_. For a reconciliation *f* in Γ(*G*, *S*), and a vertex *u* ∈ *V°*(*G*) with *f*(*u*) ≠ *r*(*S*), *f*[*u*] is a mapping defined as:

For an internal vertex *u* with *u* ≠ *r*(*G*), *f*[*u*] is a reconciliation if and only if *f*(*u*) <*f*(*p*(*u*))*;* for the root *r*(*G*) of *G*, *f*[*r*(*G*)] is a reconciliation if and only if *f*(*r*(*G*)) <*r*(*S*). In both cases, we will say that the reconciliation *f*[*u*] is obtained from *f* by applying a Nearest Mapping Change (NMC) operator on *u*; this operator is adapted from the one introduced in [20]. Similarly, we can define *f*[*u*_1_,*⋯* ,*u_k_*] for a sequence of (not necessarily distinct) vertices in G. Note that for a reconciliation *f* in Γ(*G*, *S*) with *f* ≠ *M*, there exists a unique sequence *u*_1_, *⋯* , *u_k_* so that *f* = *M*[*u*_1_, *⋯* , *u_k_*] and id(*u_i_*) ≤ id(*u_i_*_+1_) for *i* = 1,..., *k –* 1; now id(*f*) is defined as id(*u_k_*), where *u_k_* is the last vertex in this sequence. For completeness, we will use the convention id(*M*) = 0. Finally, for a reconciliation *f* in Γ(*G*, *S*), we set:

where *K*(*f*) will be regarded as an ordered list (with the order induced by id).

The NMC operator induces a tree structure on the set Γ(*G*, *S*): the root is *M*; *f′* is a child of *f* if and only if *f′* = *f*[*u*] for some *u* ∈ *K*(*f*). This tree, whose vertex set is Γ(*G*, *S*), will be denoted by *T*(*G*, *S*). The idea of considering a tree structure on the space of reconciliation was introduced in [20]. Clearly, by Theorem 2, the restriction of *T*(*G*, *S*) on Γ*_ε_*(*G*, *S*, gd) is a subtree, which will be referred to as *T_ε_*(*G*, *S*, gd). Now we can state our algorithm as follows, which enumerates Γ*_ε_*(*G*, *S*, gd) by a traversal of *T_ε_*(*G*, *S*, gd). Here ⊔ stands for disjoint union.

To see the running time of the above algorithm, note first that for a reconciliation *f*, *K*(*f*) is a subset of *V°*(*G*), and for each *u* ∈ *V°*(*G*), whether *u* ∈ *K*(*f*) or not can be determined in constant time, when id(*u*) and id(*f*) are known. In addition, if *δ_M_* is given, then line 5a and 5b can be computed in constant time; the proof of this observation will be presented in the full version of this paper. Therefore, the above algorithm runs in time *O*(|*V*(*G*)| . |Γ*_ε_*(*G*, *S*, gd)|), plus additional preprocessing time to compute id(*u*) and *δ_M_*(*u*) for each *u* ∈ *V°*(*G*).

Two facts prevent us from designing better algorithm for the enumeration problems. The first one concerns the boundary set *B_ε_*(*G*, *S*, gd), which consists of all reconciliation *f* in Γ(*G*, *S*) *–* Γ*_ε_*(*G*, S, gd) such that for some f* ∈ Γ*_ε_*(*G*, *S*, gd), f is a child of f* in *T*(*G*, *S*). In order to enumerate Γ*_ε_*(*G*, *S*, gd), an algorithm typically needs to visit not only the reconciliations in Γ*_ε_*(*G*, *S*, gd), but also those in *B_ε_*(*G*, *S*, gd). However, *|B_ε_*(*G*, *S*, gd)| could be as large as *O*(|*V*(*G*)| . |Γ*_ε_*(*G*, *S*, gd)|). For instance, if *G* and *S* have the same tree structure on *n* +1 leaves, then Γ_0_(*G*, *S*, gd) = {*M*} but *|B*_0_(*G*, *S*, gd)| contains *n –* 1 reconciliations. Furthermore, we have |Γ_1_(*G*, *S*, gd)| = *n* and *|B*_1_(*G*, *S*, gd)| = Θ(*n*^2^).

The other concern is about the set *K_ε_*(*f*) *:*= {*u* ∈ *V*(*G*) : *f*[*u*] is in Γ_ε_(*G*, *S*, gd) and id(*u*) ≥ id(*f*)}, which is needed if we want to explore Γ*_ε_*(*G*, *S*, gd) without visiting the boundary set *B_ε_*(*G*, *S*, gd). However, some properties of these two sets, *K*(*f*) and *K_ε_*(*f*), are different. For instance, the following property of *K*(*f*) is crucial to the optimal algorithm for exploring Γ(*G*, *S*) (see Property 5 and Proposition 4 in [20]): If *u* is the first vertex in *K*(*f*) and *f′* = *f*[*u*], then we have *K*(*f*) *– K*(*f*′) ⊆ {*u*}. However, this does not hold for *K_ε_*. To see it, considering the example mentioned in the previous paragraph, and denoting the first child of *r*(*G*) by *r*_1_, then we have *K*_1_(*M*) = *V°*(*G*) *–* {*r*(*G*)} while *K*_1_(*M*[*r*_1_]) = ∅.

Since Γ_0_(*G*, *S*, gd) contains the gd-optimal reconciliations, the above algorithm also provides a method for enumerating all the optimal reconciliations between a gene tree and a species tree. Since *T_ε_*(*G*, *S*, gl), as well as *T_ε_*(*G*, *S*, dc), is also a subtree of *T*(*G*, *S*), we also remark that it can be modified to list nearly-optimal reconciliations with respect to the gene loss or deep coalescence cost. Due to the limited space, the details of these algorithms are omitted here and one is referred to the full version of this work appearing in our personal website. As our on-going work, the algorithms presented here will be coded in C++ and evaluated by comparing them with the existing ones on simulation data.

## Conclusions

To investigate all reconciliations between a gene tree and a species tree, we have generalized the LCA reconciliation to define an arbitrary reconciliation as a vertex mapping from the gene tree to the species tree. This provides a new framework for investigating various mathematical issues of the reconciliation space. It allows us to give a unified approach to study reconciliations with each of the cost models. As applications, we show that the LCA reconciliation is the unique one having the smallest deep coalescence cost, and present a characterization of the reconciliations with the minimum gene duplication cost; we also develop efficient algorithms to enumerate nearly-optimal reconciliations with each cost models. In future, we shall incorporate other evolutionary forces behind the gene tree heterogeneity, such as horizontal gene transfer and recombination, into this framework.

## Competing Interests

The authors declare that they have no competing interests.

## Authors' contributions

Both authors carried out the research and drafted the paper.

## References

[B1] Metzker M Sequencing technologies - the next generation Nature Reviews Genetics 2010 11 31 46 10.1038/nrg2626 19997069

[B2] Pamilo P Nei M Relationship between gene trees and species trees Mol. Biol. Evol 1988 5 568 583 3193878 10.1093/oxfordjournals.molbev.a040517

[B3] Maddison W Gene trees in species trees Syst. Biol 1997 46 523 536 10.1093/sysbio/46.3.523

[B4] Goodman M Czelusniak J Moore G Romero-Herrera A Matsuda G Fitting the gene lineage into its species lineage: A parsimony strategy illustrated by cladograms constructed from globin sequences Syst. Zool 1979 28 132 168 10.2307/2412519

[B5] Page R Maps between trees and cladistic analysis of historical associations among genes, organisms, and areas Syst. Biol 1994 43 58 77

[B6] Ma B Li M Zhang L From gene trees to species trees SIAM J. Comput 2010 30 729 752

[B7] Zhang L From gene trees to species trees II: species tree inference by minimizing deep coalescence event IEEE/ACM Transactions on Computational Biology and Bioinformatics 2011 accepted 10.1109/TCBB.2011.83 21576759

[B8] Bonizzoni P Della Vedova G Dondi R Reconciling a gene tree to a species tree under the duplication cost model Theoretical Computer Science 2005 347 36 53 10.1016/j.tcs.2005.05.016

[B9] Gorecki P Tiuryn J DLS-trees: A model of evolutionary scenario Theoretical Computer Science 2006 359 378 399 10.1016/j.tcs.2006.05.019

[B10] Arvestad L Lagergren J Sennblad B The gene evolution model and computing its associated probabilities J. ACM 2009 56 2 1 44

[B11] Chen K Durand D Farach-Colton M Notung: A program for dating gene duplications and optimizing gene family trees Journal of Computational Biology 2000 7 429 447 10.1089/106652700750050871 11108472

[B12] Eulenstein O Mirkin B Vingron M Duplication-based measures of difference between gene and species trees Journal of Computational Biology 1998 5 135 148 10.1089/cmb.1998.5.135 9541877

[B13] Bansal M Eulenstein O The multiple gene duplication problem revisited Bioinformatics 2008 23 132 138 10.1093/bioinformatics/btn150 PMC2718628 18586705

[B14] Liu L Yu L Kubatko L Pearl D Edwards S Coalescent methods for estimating phylogenetic trees Mol. Phylogenet. Evol 2009 53 320 328 10.1016/j.ympev.2009.05.033 19501178

[B15] Chauve C El-Mabrouk N Batzoglou S New perspectives on gene family evolution: Losses in reconciliation and a link with supertrees Research in Computational Molecular Biology, LNCS 5541 2009 Springer Berlin / Heidelberg 46 58

[B16] Degnan J Rosenberg N Gene tree discordance, phylogenetic inference, and the multispecies coalescent Trends in Ecology and Evolution 2009 24 332 340 10.1016/j.tree.2009.01.009 19307040

[B17] Than C Rosenberg N Consistency properties of species tree inference by minimizing deep coalescences Journal of Computational Biology 2011 18 1 15 10.1089/cmb.2010.0102 21210728

[B18] Than C Nakhleh L Species tree inference by minimizing deep coalescences PLoS Computational Biology 2009 5 9 e1000501 10.1371/journal.pcbi.1000501 19749978 PMC2729383

[B19] Kingman J Origins of the coalescent. 1974-1982 Genetics 2000 156 1461 1463 11102348 10.1093/genetics/156.4.1461 PMC1461350

[B20] Doyon J Chauve C Hamel S Space of gene/species trees reconciliations and parsimonious models Journal of Computational Biology 2009 16 1399 1418 10.1089/cmb.2009.0095 19754270

[B21] Doyon J Hamel S Chauve C An efficient method for exploring the space of gene tree/species tree reconciliations in a probabilistic frameworkpreprint 2010 10.1109/TCBB.2011.64 21464510

[B22] Arvestad L Berglund A Lagergren J Sennblad B Gene tree reconstruction and orthology analysis based on an integrated model for duplications and sequence evolution Proceedings of the eighth annual international conference on Research in computational molecular biology, RECOMB ’04 2004 326 335

